# Comparison of Monkeypox Viruses Pathogenesis in Mice by *In Vivo* Imaging

**DOI:** 10.1371/journal.pone.0006592

**Published:** 2009-08-11

**Authors:** Jorge E. Osorio, Keith P. Iams, Carol U. Meteyer, Tonie E. Rocke

**Affiliations:** 1 Department of Pathobiological Sciences, School of Veterinary Medicine, University of Wisconsin, Madison, Wisconsin, United States of America; 2 U. S. Geological Survey-National Wildlife Health Center, Madison, Wisconsin, United States of America; U.S. Naval Medical Research Center Detachment/Centers for Disease Control, United States of America

## Abstract

Monkeypox viruses (MPXV) cause human monkeypox, a zoonotic smallpox-like disease endemic to Africa, and are of worldwide public health and biodefense concern. Using viruses from the Congo (MPXV-2003-Congo-358) and West African (MPXV-2003-USA-044) clades, we constructed recombinant viruses that express the luciferase gene (MPXV-Congo/Luc+and MPXV-USA-Luc+) and compared their viral infection in mice by biophotonic imaging. BALB/c mice became infected by both MPXV clades, but they recovered and cleared the infection within 10 days post-infection (PI). However, infection in severe combined immune deficient (SCID) BALB/c mice resulted in 100% lethality. Intraperitoneal (IP) injection of both MPXV-Congo and MPXV-Congo/Luc+resulted in a systemic clinical disease and the same mean time-to-death at 9 (±0) days post-infection. Likewise, IP injection of SCID-BALB/c mice with MPXV-USA or the MPXV-USA-Luc+, resulted in similar disease but longer (P<0.05) mean time-to-death (11±0 days) for both viruses compared to the Congo strains. Imaging studies in SCID mice showed luminescence in the abdomen within 24 hours PI with subsequent spread elsewhere. Animals infected with the MPXV-USA/Luc+had less intense luminescence in tissues than those inoculated with MPXV-Congo/Luc+, and systemic spread of the MPXV-USA/Luc+virus occurred approximately two days later than the MPXV-Congo/Luc+. The ovary was an important target for viral replication as evidenced by the high viral titers and immunohistochemistry. These studies demonstrate the suitability of a mouse model and biophotonic imaging to compare the disease progression and tissue tropism of MPX viruses.

## Introduction

Human monkeypox (MPX) is a zoonotic viral exanthema with manifestations similar but less severe than smallpox [1]. The virus (MPXV) belongs to the *Orthopoxvirus* genus of the *Poxviridae* family and shares many biochemical and physical properties with other orthopoxviruses, such as vaccinia and variola. MPXV is thought to be maintained in wild rodents in the rain forests of Central and West Africa, causing sporadic human outbreaks in remote villages probably as a result of direct cutaneous contact or mucosal exposure to infected animals [2]–[5]. Because the airborne route of exposure is known to play a role in secondary human-to-human transmission [6], concerns have been raised about the potential use of MPX as a biological warfare agent and as such, the virus is listed as a Category C select agent.

Monkeypox emerged for the first time in the Western Hemisphere in 2003, causing an outbreak in the Midwestern United States affecting 37 people that were exposed to ill prairie dogs purchased from pet stores or through pet swaps [7]–[10]. The virus entered the US upon the importation of exotic rodents from Ghana (West Africa). Subsequent studies demonstrated the existence of two genetically distinct variants of the virus, called the West African and Congo Basin clades [11]. The strain that caused the US outbreak belonged to the West African clade; which is associated with less severe disease as compared to the Congo Basin clade [12].

Several animal models have been used to study MPXV pathogenesis, including newborn mice and rats [13], cynomolgus monkeys [14], [15], squirrels [16], [17], prairie dogs [9], [18], and dormice [19]. Some of these studies were conducted using conventional methods involving large sample size and sacrificing animals to determine viral titers and histological changes. In the present study, we describe the development of recombinant MPXV expressing the luciferase gene (MPXV-USA-Luc+, MPXV-Congo/Luc) and their use in monitoring disease progression *in vivo* with biophotonic imaging. This technique has been used to study a variety of bacterial and viral infections [20]–[24]. Biophotonic imaging offers significant advantages over conventional pathogenesis studies because it can: 1) be used to quantitatively visualize viral infections in living animals; 2) allow disease progression and outcome to be directly linked to virus replication and virus load; 3) provide significant ethical advantages because experiments can be carried out with fewer animals; 4) result in faster data acquisition since images can be quantified within minutes; and 5) reveal unsuspected sites of viral replication and modes of viral spread.

Using luminescent MPX viruses, we compared disease progression in both immunocompetent and immuno-compromised mice between the West African and Congo clades via IP exposure. This system could be used to address many questions about MPX pathogenesis, including virulence factors, disease progression in rodent hosts, and viral shedding from infected animals, an index of the transmission potential to humans and other animals. In addition, these tools can be used to test anti-virals and the next generation of orthopoxvirus vaccines for their ability to alter the course of disease.

## Results

### Generation of recombinant virus and one-step growth curves of MPXV

**Figure 1:**
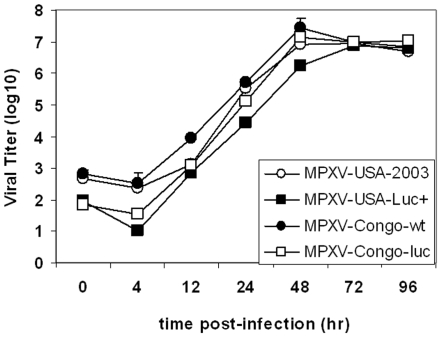
One-step growth curves for parental (MPXV-Congo, MPXV-USA-2003) and progeny recombinant (MPXV-Congo-Luc+, MPXV-USA-Luc+) viruses. Vero cell monolayers were infected at multiplicity of infection (MOI) of 0.1 with parental (MPXV-Congo, MPXV-USA-2003) or with progeny (MPXV-Congo-Luc+, MPXV-USA-Luc+) strains. After allowing for attachment (30 min), cells were washed twice with PBS to remove unattached virus. Then fresh medium as added and plates were incubated at 37°C 5% CO2. At various intervals thereafter, three wells per virus strain were harvested (media and cells) and placed at −70°C. After three cycles of freezing and thawing, the samples were sonicated and virus titers were determined by serial dilution and infection of Vero cell monolayers. Plaques were visualized by staining with 0.1% crystal violet in 20% ethanol and virus titers determined as described elsewhere [39].

Sequencing and PCR analysis showed that recombinant MPXV-USA-Luc+and MPXV-Congo-Luc+contained the luciferase gene inserted into the 176–177 intergenic regions. To determine whether this insertion adversely affected the overall growth characteristics of MPX viruses, we carried out one-step growth experiments in Vero cell monolayers. Total virus production, expressed as PFU/ml, was determined for samples collected at various times after infection. For both wt and Luc+viruses, the lag and rise period of exponential growth were of similar duration, and gave comparable yields (Figure 1). Thus, within experimental limitations, we concluded that the insertion of the luciferase gene in our engineered viruses did not limit growth in Vero cells.

### Clinical presentation, morbidity, and mortality in mice

Several experiments were conducted to evaluate a mouse model for MPXV infection. First, four-week-old BALB/c mice (n = 4) were exposed to either wt or recombinant Luc+MPXV by the IP route and monitored for clinical signs. Infected mice exhibited rough coat, inappetence, and decreased activity within 5 days and recovered by approximately 10 days post-infection (dpi). A group of uninfected controls (4 animals) did not develop any signs of disease.

In order to more fully characterize viral pathogenesis and compare the virulence of the MPXV-USA-Luc+and MPXV-Congo-Luc+strains with parental viruses, the next set of experiments was conducted in 4-week-old immunocompromised SCID-BALB/c mice. Groups of four SCID-BALB/c mice were IP inoculated with 10^5^ plaque forming units (PFU) with either the recombinant MPXV-USA-Luc+or MPXV-Congo-Luc+strains, the wild-type MPXV-USA or MPXV-Congo strains, or diluent (n = 2) and monitored for clinical signs. Within 5 DPI, both MPXV-Congo and MPXV-Congo/Luc+inoculated groups had evident signs of a systemic clinical disease (rough coat, inappetence, decreased activity). All mice in both groups died on the same day (day 9), indicating that insertion of the Luc+gene did not result in viral attenuation. Inoculation of MPXV-USA and MPXV-USA/Luc+viruses in SCID mice produced a similar disease, but clinical signs were not observed until 7 DPI and all animals inoculated with both strains died on the same day (day 11). The mean time-to-death of MPXV-USA strains was significantly longer (P<0.05) than the MPXV-Congo strains, indicating that the West African MPXV clade is less pathogenic. Mice that received diluent remained healthy.

### Visualization of MPXV infection

**Figure 2:**
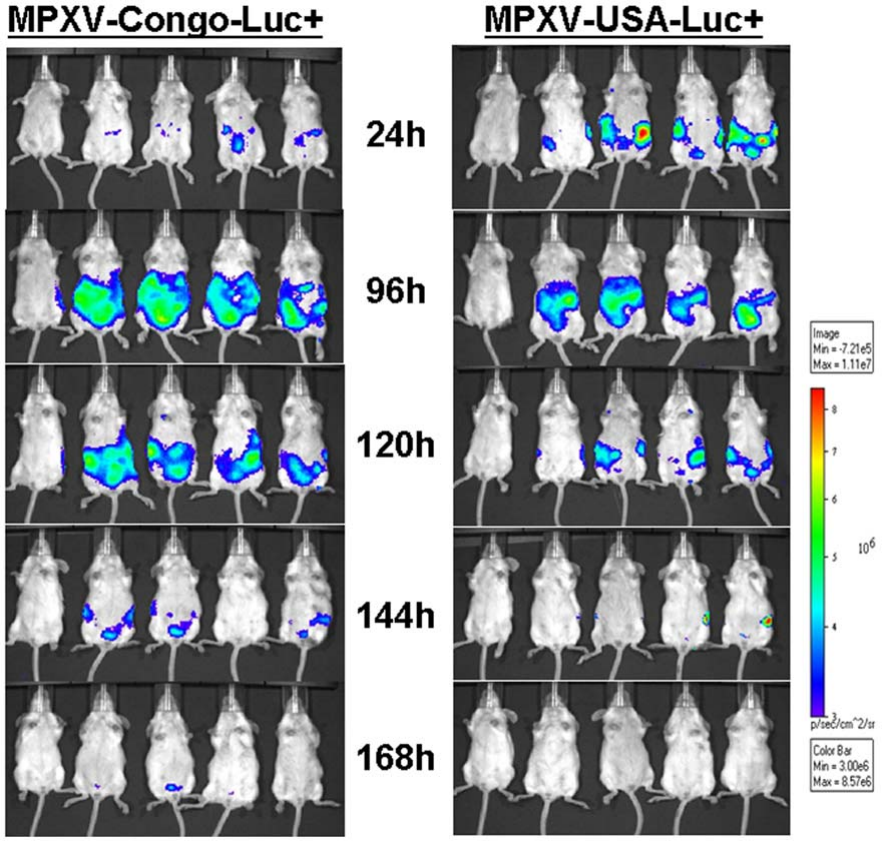
In vivo imaging of MPXV-Congo-Luc+ (left panel) and MPXV-USA-Luc+ (right panel) in BALB/c mice (Intraperitoneal inoculation). Groups of four, 4-week-old BALB/c mice were inoculated by the IP route with 105 PFU of either MPXV-Congo-Luc+or MPXV-USA-Luc+viruses. At indicated times post-infection, mice were injected IP with 1.5 mg luciferin in 100 µl of DPBS (Promega, Madison, WI) and imaged (ventral view) in an IVIS 200 imager (Caliper Life Sciences, Alameda, CA). Exposures for 30 sec (F8, medium binning) were taken at approximately 12 minutes post-luciferin injection following anaesthetization with isoflurane. An uninfected negative control animal (first animal on left side) was also injected with luciferin and imaged on the same times as infected animals. Images were analyzed with Living Image 3.0 software (Caliper Life Sciences, Alameda, CA). A) 24 h; B) 96 h; C)168 h; D) 240 h.

The MPXV-USA-Luc+and MPXV-Congo-Luc+strains were used to monitor viral infection *in vivo* with biophotonic imaging. Twenty-four hours after IP inoculation of the recombinant virus and every day afterwards, SCID and immunocompetent BALB/c mice were injected IP with luciferin and placed in the imager. In BALB/c mice, luminescent signal was visualized as early as 24 hours PI (Figure 2). Infection with the MPXV-Congo-Luc+produced a more intense signal than MPXV-USA-Luc+, suggesting stronger replication and faster spread. This signal peaked between 96-120 hours PI and was mostly limited to the organs in the peritoneal cavity, with occasional spread to the axillary lymph nodes. For both viruses, luminescent signal was undetectable by 240 hours, indicating that these animals had cleared the infection.

**Figure 3:**
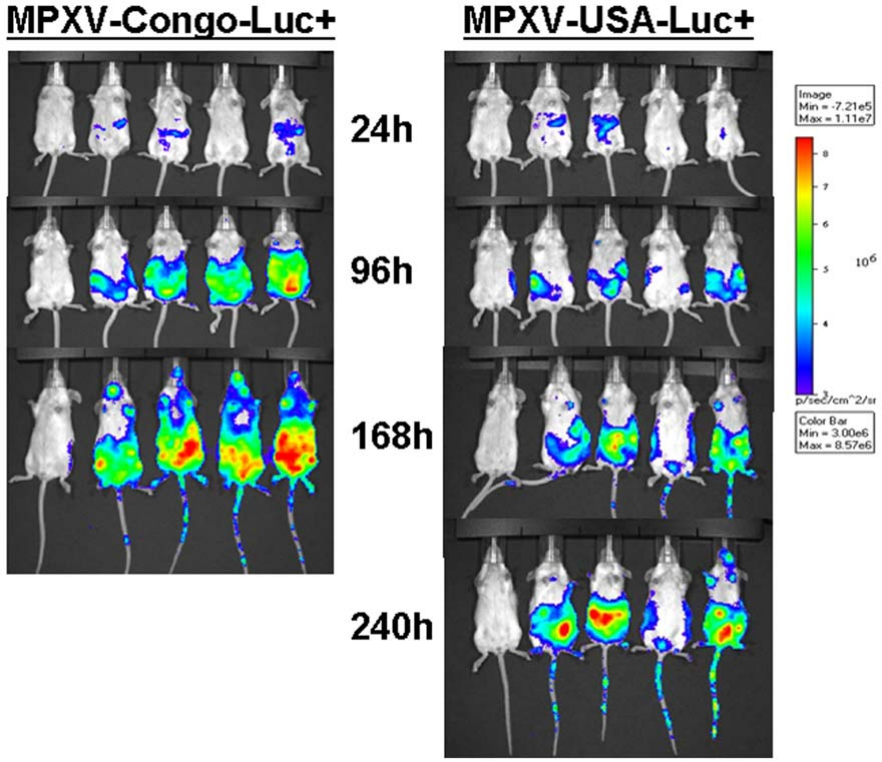
In vivo imaging of MPXV-Congo-Luc+ (left panel) and MPXV-USA-Luc+ (right panel) in SCID- BALB/c mice (Intraperitoneal inoculation). A group of four, 4-week-old SCID BALB/c mice was inoculated by the IP route with 105 PFU of MPXV-USA-Luc+virus and imaged as described as described in materials and methods. An uninfected negative control animal (on left) was also injected with luciferin and imaged on the same times as infected animals. Ventral view.

In SCID- BALB/c mice, luminescence indicative of MPXV infection was also visible as early as 24 hours PI and limited at that time to the peritoneal cavity (Figure 3). Once again, infection with the MPXV-Congo-Luc+produced a more intense luminescent signal, and by 96 hours PI, it had spread to other organs and tissues in the abdominal region, the thoracic area, and axillary lymph nodes. At 168 hours PI, luminescent signal was detected in the entire body and animals died between 192 and 216 hours PI. In SCID mice inoculated with MPXV-USA/Luc+, luminescent signal was also visible in the abdominal region at 96 hours PI, and it was visible in the tail, feet, and nasal area at 240 hours PI. All of these animals died by 264 hours PI.

### Virus titers from selected tissues and correlation with luminescence

**Figure 4:**
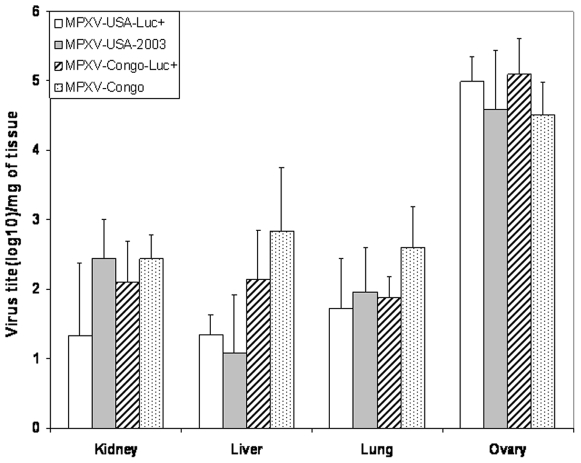
Virus titers from selected tissues and correlation with luminescence. Tissues samples from kidney, liver, lung and ovaries were aseptically harvested to compare viral titers between the parental MPXV-USA-2003 and recombinant progeny MPXV-USA-Luc+strains. Tissue homogenates were centrifuged as described in materials and methods section. Viral titers were calculated per gram of tissue.

**Figure 5:**
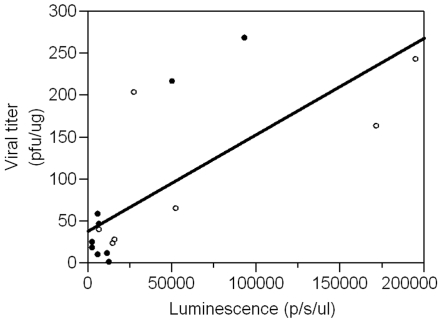
Correlation between viral titers with luminescence levels. The luminescence of kidney, liver, and lung tissue lysates was measured with the IVIS imager. A calibration curve was then generated using inverse regression analysis and plotting virus titer (PFU/g) against luminescence (photons/sec). MPXV-USA-2003-Luc+(•). MPXV-Congo-Luc+(○).

To monitor viral titers, tissues were aseptically harvested at the time of death to compare viral titers between the parental viruses and recombinant progeny Luc+strains and to correlate titers with luminescence levels. No differences in viral titer were detected for the Congo Luc+and wt strains for kidney (P = 0.49), liver (P = 0.22), lung (P = 0.25) and ovary (P = 0.60). Likewise, no differences in viral titer were detected between animals infected with the USA/Luc+and wt strains (Figure 4) for kidney (P = 0.41), liver (P = 0.75), lung (P = 0.68), and ovary (P = 0.89). These results provide further support that insertion of the luciferase gene did not substantially alter virulence of the virus. Viral titers in the ovaries were about 2 logs higher than in other tissues for both the Congo and USA strains. Using data collected from kidney, liver and lung extracts from both MPXV-Luc+strains, a correlation (Figure 5) was detected between measured luminescence and viral titer (R^2^ = 54%; P = 0.0008). Data from ovarian extracts were not included in the analysis because viral titers were much higher than the other tissues. With the resulting calibration curve generated, approximate viral titer can be calculated in future studies using the following formula: titer = 38.587+0.0011 photons/s/µl.

### Immunostaining of tissues derived from MPXV infected animals

**Figure 6:**
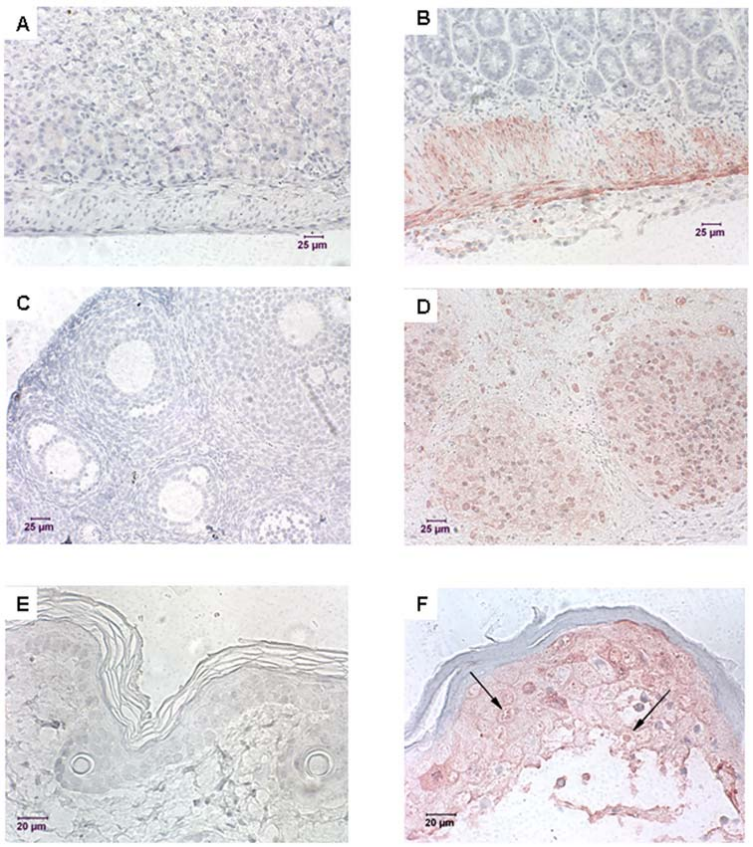
Immuno-histochemical staining in tissues of mice infected with MPXV. Tissues from 4-wk-old SCID/BALB/c mice infected IP with MPXV-USA-2003-Luc+and uninfected SCID/BALB/c mice were stained by using vaccinia mouse hyperimmune mouse and horse-radish peroxidase as a detection label. MPXV antigen was identified in the intestine, ovary and skin of the feet (7B, D, and F respectively). Poxviral inclusions were seen in the skin of a foot (7F arrows). MPXV antigen was also found in the nasal turbinate of IN infected mice (7H). None of the IHC-stained tissues of the control mice including intestine, ovary, and skin (7A, C, and E respectively) had viral antigen staining.

MPXV antigen was consistently detected in ovary, intestinal muscle wall and skin of the feet sampled from SCID/BALB/c mice inoculated IP with either parental (MPXV-Congo, MPXV-USA-2003) or recombinant (MPXV-Congo-Luc+, MPXV-USA-Luc+) viruses (Figure 6B, D, and F). In addition, small random areas containing MPXV antigen were detected in lung, heart, liver, kidney, and pancreas (data not shown). No MPXV antigen was detected in any of the tissues sampled from uninfected SCID/BALB/c mice (Figure 6A, C, and E). The amount of antigen staining in the ovary was diffuse with intense antigen staining of follicular tissue.

### Histologic Findings

**Figure 7:**
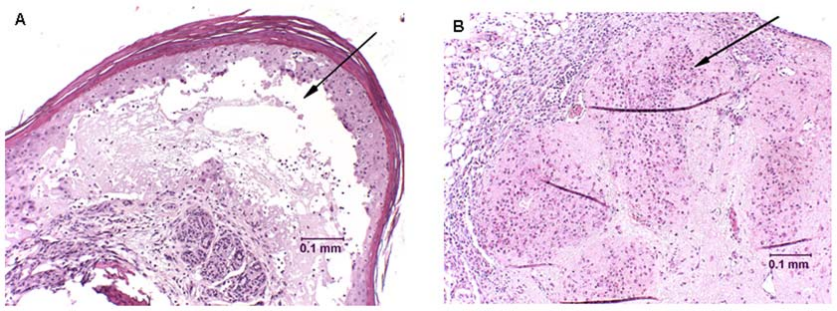
Histological sections from mice infected with MPXV-USA-2003 stained with hematoxylin and eosin. A) Necrosis of ovarian follicles (arrow) with subacute inflammation infiltrating surrounding connective tissue and peri-ovarian fat. B) Skin of foot with intradermal bulla containing edema and cell debris (arrow). Surrounding epidermis is undergoing ballooning degeneration.

Consistent pathology was seen in the ovary, skin, and serosa of intestine sampled from SCID BALB/c mice infected IP with wt parental and recombinant MPXV-USA-2003 and MPXV-Congo clades. The ovary was severely necrotic with loss of architecture and subacute inflammation of surrounding tissues (Figure 7A). The wall of the ovarian bursa was thickened with neutrophils, macrophages and necrotic debris. Neutrophils, macrophages and red blood cells were also present in the open space of the ovarian bursa. The serosa of the intestine was mildly proliferative and occasionally associated necrosis of underlying smooth muscle. Multifocal lesions involving the skin of the feet and tail consisted of hyperkeratosis, acanthosis and subacute deep dermal inflammation. Eosinophilic cytoplasmic inclusion bodies, suggesting viral inclusions (Guarnieri bodies), were infrequently present in the vacuolated epithelium of the stratum spinosum of the hyperkeratotic skin (Fig. 6F). Intradermal bullae were filled with edema and scattered necrotic debris (Figure 7B) with ballooning degeneration of surrounding epithelium. Mild multifocal apoptosis was less consistently seen in liver and pancreas (data not shown).

## Discussion

By constructing recombinant MPX viruses that expresses the luciferase gene (Luc+), we have characterized and compared the progression of disease in mice infected with MPXV-Congo and MPXV-USA strains. In addition, we established appropriate animal models for further study of MPX viruses in general. We found that 4-week-old immunocompetent BALB/c mice became ill after IP exposure to either the Congo or USA viruses but recovered fairly quickly from the infection. In contrast, 4-week-old immunocompromised SCID-BALB/c mice were highly susceptible to MPXV, with infection resulting in 100% lethality for both the Congo and USA viruses. Time-to-death in SCID BALB/c mice following IP infection was similar for the parental wild type (wt) and recombinant Luc+viruses for both the Congo and USA strains, indicating that insertion of the Luc+gene did not result in viral attenuation, but the Congo viruses have stronger replication and faster spread confirming previous reports regarding the increased virulence of this viral clade [25]. Our studies are the first of this type to provide an extensive evaluation of MPXV infection and disease progression in well defined mouse laboratory strains. Although previous work reported MPXV infection in newborn laboratory rats and mice [26], the genetic background of the animals used in that study were not described.

The use of a recombinant MPX- Luc+viruses and biophotonic imaging provided significant advantages over conventional pathogenesis experiments involving tissue harvesting and titration studies to determine MPX disease progression and its correlation to virus replication/luminescence levels in the laboratory mouse model. While the limit of detection by luminescence for our MPX-Luc+viruses is unknown, previous studies with Sindbis virus have shown to be approximately 10^3^ PFU/g [23]. Subsequent studies will focus in the validation of *in vivo* imaging through serial sacrifice studies and comparison of luminescence and viral levels in organs throughout the disease course. Following IP inoculation of SCID mice, luminescence-indicative of MPXV-Luc+replication-was visible in the peritoneal cavity within 24 hours PI, and during early stages, the infection for both viral clades was limited to organs in the abdominal region. Infection with MPXV-Congo-Luc+spread faster and by 96 hours was detected in lymph nodes in the axilliary region, whereas for MPXV-USA-Luc+only later (day 7-10) was luminescent signal visible in the nasal area, tail and feet. It is unclear whether spread to these areas occurred following viremia facilitated by infected dendritic cells or through viral shedding in feces or urine. For other poxviruses, such as ectromelia, the skin is the primary site of viral infection [27].

*In vivo* imaging of SCID mice injected IP revealed a very high tropism of MPXV for ovarian tissues. This result was confirmed by the high viral titers (>10^5^ PFU) measured in ovaries of infected mice and IHC studies that showed ovaries had the most intense and diffuse staining compared to other tissues. A previous study in non-human primates also reported detection of MPXV antigen in ovarian tissues [14], but it was not a primary site of viral replication. The extent of viral spread in SCID BALB/c mice was affected by the viral clade. Inoculation by the IP route of both viral clades resulted initially in infection of organs in the peritoneal cavity. Then, for MPXV-Congo-Luc+, infection resulted in a more disseminated spread and luminescence signal was detected in the entire body.

While these studies provide new knowledge regarding the pathogenesis of MPXV in the laboratory mouse, the relevance of this model when compared to human monkeypox disease and non-human primate model remains to be seen. Following IP inoculation, mice developed a systemic disease and virus was detected in multiple organs, including lungs, kidneys and ovaries. Furthermore, *in vivo* imaging showed significant viral replication in the skin (tail, feet), producing multiple well-defined pustule lesions with the presence of Guarneri inclusion bodies as confirmed by histology and immunhistochemistry. However, no clinical signs of rash were observed in infected animals, suggesting that this model might not be completely comparable to human monkeypox disease. The IP route of inoculation was used in this initial study in order to provide a highly consistent dose of virus to establish imaging procedures. Parenteral routes of infection (intravenous, subcutaneous, intraperitoneal, footpad) have been used by others to establish MPXV animal models in non-human primates, squirrels, prairie dogs and dormice [16], [18], [28], [29]. In future studies, the intranasal route will be used since this route simulates natural infection with MPXV.

The marked difference in pathogenesis observed between SCID mice and immune-competent BALB/c mice provides an opportunity to investigate the immune responses that protect against MPXV infection. Because luminescence in the immunocompetent BALB/c mice peaked at 96 hours post-infection, early events in the host immune response are probably important in controlling MPXV infection. While SCID mice lack of T or B cell responses, they can fully mount innate (e.g cytokines) immune responses. In subsequent studies we will compare these innate responses between immunocompetent and SCID BALB/c mice in an attempt to elucidate their role in MPXV infection. Studies with vaccinia have demonstrated the importance of interferon in viral spread and pathogenesis since IN infection in mice lacking receptors for type I interferons (IFN I R −/−) resulted in more systemic spread into abdominal organs [30]. There is little consensus at the present about the correlates of protection in animals infected with MPXV or other related poxviruses. Most of the understanding of the host response to poxvirus infection in humans comes from historical clinical data collected from smallpox patients and vaccinated individuals. Cytotoxic T lymphocytes (CTL) and antibody responses are associated with virus control in vaccinia-vaccinated individuals and those who have previously recovered from smallpox [31]. Patients with abnormalities in T-cell function developed generalized vaccinia, whereas patients with congenital agammaglobulinemia did not [32]. There is also a growing appreciation of the importance of antibody in virus control and animal recovery in other models of both primary and secondary poxvirus infections. In macaques, vaccinia vaccination induced protection against a lethal intravenous challenge with MPXV [33]. Animals depleted of B cells were susceptible to infection, but not if they were depleted of either CD4 or CD8 T cells [34].

In addition to their use for the study of MPXV pathogenesis, these luciferase-expressing viruses in combination with *in vivo* animal models can also provide important tools in the development of novel anti-orthopoxvirus therapeutics. Similar studies have been conducted using luminescent herpes simplex virus type 1 (HSV-1) [21]. No antiviral drug has been proven to be effective in the treatment of human smallpox. The only antiviral agent currently approved for use against orthopoxviruses is cidofovir [35]. However, this compound has low oral bioavailability and must be administered intravenously, limiting its usefulness.

The recombinant MPXV-Luc+viruses we constructed appear to be highly stable and fully virulent. After sequence analyses, we selected several MPXV intergenic regions, including 141–142 and 176–177, as the sites for Luc+insertion. The primary factors involved in the selection of these regions included the lack of potential promoter sequences and also sufficient distance from nearby genes, thus decreasing the chance of functional disruption by the foreign gene insertion. Although we initially inserted the luciferase gene into the 141–142 region, the resulting virus had reduced virulence compared to the parental virus in SCID mice (data not shown). The recombinant MPXV-Luc+used in this study was created using the 176–177 intergenic region. Following five rounds of plaque purification, and pathogenesis studies in 12 animals, the luciferase insert was still present in the recombinant viruses as shown by luminescence and sequence data, indicating that the MPXV-Luc+were stable and the 176–177 intergenic region can efficiently maintain the foreign gene. In addition, i*n vitro* experiments, such as one-step growth curves, and *in vivo* virulence studies in mice showed that recombinant Luc+viruses maintained the phenotypic and virulence properties of the parental viruses.

Another advantage of the Luc+insertion is the ability to use the marker to quantify virus in animal tissues. We found a strong correlation between luminescence in tissue extracts from infected mice and viral titers quantified by traditional plaque assays. This finding can be used in future studies for faster quantification of virus loads, avoiding the significant biohazard involved in harvesting and processing tissues for viral titration, particularly for select agents such as MPXV. Unfortunately, quantification of virus using this method is not as sensitive as plaque assays and molecular techniques (real time PCR). However, the ability to monitor animals longitudinally compensates for the loss in sensitivity compared to that of the plaque assay and adds the dimension of time to disease progression and pathogenicity studies.

In summary, we have constructed highly stable recombinant MPXV- Luc+viruses that can be used in biophotonic imaging studies to provide further insight into MPXV pathogenesis and host response to infection. The availability of these viruses also provide a unique opportunity to study MPXV infection in known wild rodent hosts from Africa as well as prairie dogs and other U.S. rodents that could serve as hosts of the virus if subsequent introductions of the virus occur. In future studies, we will use luciferase-expressing viruses to study pathogenesis via IN and other routes of infection and to better assess the role of specific genes in the pathogenesis of MPXV. A recent study suggested that several genes, including D10L, D14L, B10R, B14R and B19R might play an important role in MPXV virulence [12]. Understanding the factors that increase MPXV virulence can aid the development of vaccines and anti-virals that could be used to prevent or treat human monkeypox.

## Materials and Methods

### Viruses and cells

MPXV-USA (Strain designation 044) was kindly provided by Dr. Inger K. Damon. (CDC, Atlanta, GA). This virus was isolated during the USA outbreak in 2003 [10]. MPXV-Congo was isolated during a 2003 outbreak of MPX in the Republic of Congo (ROC) and designated as MPXV-2003-358. Recombinant viruses were generated and amplified on cell monolayers of rat embryonic fibroblasts (Rat-2, CRL-1764) or African green monkey kidney epithelial cells (BSC-1: CCL-26; or Vero: CCL-18) obtained from American Type Culture Collection (ATCC), Manassas, VA. Cell cultures were maintained at 37°C and 5% CO_2_ in Medium 199 supplemented with 0.01 g/L L-glutamine and 5% fetal bovine serum (FBS).

### Construction of pGPT/luc/PCSII recombinant plasmid vector

We used the guanine phosphoribosyl transferase (GPT) gene as a selection system to generate recombinant MPXV containing the luciferase (Luc+) marker. For this purpose, we constructed a plasmid (pGPT/luc/PCSII) containing the GPT and Luc+genes under the control of the synthetic early late promoter (SE/L). This plasmid also contains MPXV sequences to allow cloning into the 176–177 intergenic regions for both MPXV-Congo and MPXV-USA clades.

### Construction of a MPXV transfer vector with polycloning sites

**Figure 8:**
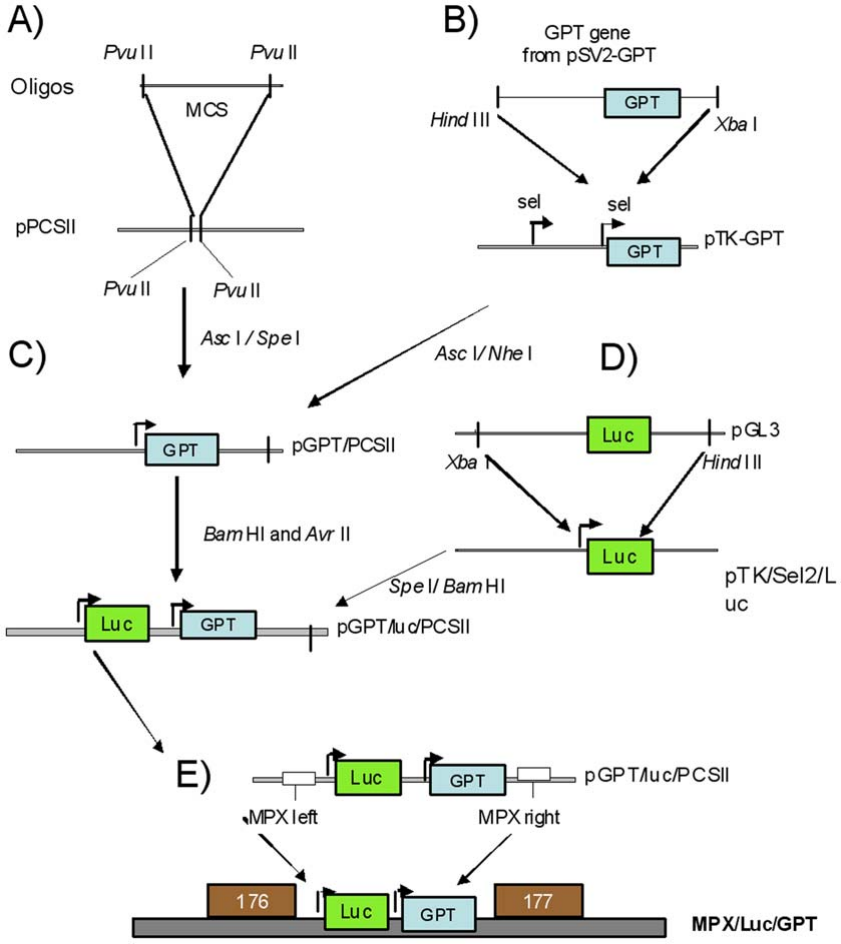
Construction of MPXV-Luc+viruses. A) Two synthetic DNA fragments containing the p7.5 promoter from vaccinia were annealed and cloned into the pUC18 plasmid, resulting into the pPCSII, plasmid. B) The GPT gene was PCR amplified and cloned into the pTK/Sel2 plasmid resulting in the pTK-GPT plasmid. C) The SE/L-GPT fragment was removed from the pTK-GPT plasmid and ligated into the pPCSII plasmid generating the pGPT/PCSII plasmid. D) The luciferase gene was cloned into the plasmid pTK/Sel2/luc plasmid. E) The SE/L-luciferase fragment was cloned into the pGPT/PCSII plasmid to generate pGPT/luc/PCSII construct. Then, MPXV regions, sequences for the left (176L) and right (176R) flanking sequences of the intergenic region 176–177 were cloned into the pGPT/luc/PCSII. The resulting plasmid was then used to generate recombinant MPXV.

Plasmid pUC18 was digested with *Pvu* II and gel-purified to remove the *LacZ* and polycloning site sequences. This plasmid contributed the backbone for the MPX transfer vector. Then, two oligonucleotides (5′-GGCCGGCCGGACCGACACCCTAGGACTAGTCGATGCTAGCGCCAGGCGCGCCGGGCCC-3′ and 5′-GGGCCCGGCGCGCCTGGCGCTAGCATCGACTAGTCCTAGGGTGTCGGTCCGGCCGGCC-3′) were synthesized and annealed to form a double stranded molecule containing multiple cloning sites. For this purpose, 2 µg of each oligonucleotide were resuspended in 100 µl of 50 mM Tris pH 8.0, and incubated at 72°C for 10 min. The mixture was then allowed to slowly cool to room temperature. Ten microliters of the annealed mixture were employed in a blunt-end ligation reaction (room temperature, overnight) with the *Pvu* II-digested pUC18 plasmid. The resulting plasmid, designated pPCSII, was sequenced and then purified for further manipulation (Figure 8A).

### Cloning of the GPT gene under poxvirus promoter control

Plasmid pSV2-GPT (ATCC #37145) was used to clone the GPT gene. Primers for GPT amplification were 5′-CGTACAT***AAGCTT***TGGGACACTTCACATGAGCG-3′ containing a *Hind* III site (bold italicized) and 5′-GTGA***TCTAGA***GACGACGGTCACTAGTGGAAACTATTGTAACCCGCC-3′ containing an *Xba* I site. The two restriction enzyme recognition sites were included to facilitate subsequent cloning procedures. Amplification was carried out for 35 cycles at 94°C for 30 sec, 50°C for 15 sec and 72°C for 2 minutes. The amplification product was purified by passage through a Qiaquick PCR purification column (Qiagen Sciences, Valencia, CA), digested with *Hind* III and *Xba* I restriction enzymes at 37°C for 3 hr, and purified by another passage through a Qiagen PCR purification column. The purified fragment was then cloned into the pTK/Sel2 plasmid [36] that had been previously digested with *Hind* III and *Xba* I. This plasmid, named pTK-GPT (Figure 8B) contains the GPT gene downstream from a poxvirus synthetic early/late promoter (SE/L).

### Construction of the MPXV transfer vector containing the GPT gene

The pTK-GPT plasmid was digested with restriction enzymes *Asc* I and *Nhe* I to excise the SE/L- GPT fragment. Simultaneously, the pPCSII plasmid was digested with *Asc* I and *Spe* I enzymes. *Spe* I generates identical overhanging ends as *Nhe* I and facilitates ligation of the *Nhe* I/*Asc* I fragment containing GPT into the vector with destruction of both *Spe* I and *Xba* I restriction enzyme sites. The resulting product was purified through a Qiaquick column and ligated to the GPT/SE/L fragment to generate the pGPT/PCSII plasmid (Figure 8C).

### Cloning of the luciferase gene under poxvirus control

To generate a luciferase gene under the control of a poxvirus promoter, we first digested plasmid pTK/Sel2 with restriction enzymes *Xba* I and *Hind* III which was purified through a Qiaquick PCR purification column. To obtain the luciferase gene, plasmid pGL3 (Promega, Madison WI) was also digested with *Xba* I and *Hind* III enzymes and the lucifierase-containing DNA fragment was purified from an agarose gel. The fragment and vector were ligated to form plasmid pTK/Sel2/luc (Figure 8D).

### Assembly of the GPT/luciferase transfer vector

The pTK/Sel2/luc plasmid was digested with *Spe* I and *Bam* HI and the SE/L-luciferase fragment was extracted from an agarose gel and purified as described above. Simultaneously, the plasmid, pGPT/PCSII was digested with *Bam* HI and *Avr* II and gel purified. Enzyme *Avr* II contains overhanging ends that are compatible with *Spe* I which facilitated the ligation of the SE/L-luciferase fragment into the pGPT/PCSII plasmid to generate pGPT/luc/PCSII construct.

To target the integration of the two GPT and Luc+genes into specific MPXV regions, sequences for the left flanking sequence (176L) of the intergenic region 176–177 from MPXV-USA-2003 strain were PCR amplified using primers 5′CCGGCGCATATGGACTTACATAAATATCTGGGA 3′ and 5′AATTCGGCCGGACCGATACGATTATTAATAGCCG-3′. The resulting PCR product was digested with *Nde* I/*Eag* I and the 316 base pair (bp)-fragment was cloned into the pGPT/luc/PCSII. The right flanking sequence (176R) was also PCR amplified using primers 5′ GCCGCTCGAGGCGATGGATTTAAACATC 3′ and 5′ TTAAGGCGCGCCGTTAAAATACATTCTAATACGG 3′ and the cDNA product was digested with *Xho* I/*Asc* I generating a 297 bp fragment that was then cloned into the pGPT/luc/PCSII. The resulting plasmid was then used to generate recombinant MPXV (Figure 8E).

### Generation of recombinant MPXV viruses

Propagation of recombinant poxviruses using GPT selection method was performed as described [37]. Briefly, BSC-1 cells at 80% confluence were infected at a multiplicity of infection (MOI) of 0.06 with wt MPXV and then transfected with 4 µg of plasmid DNA mixed with10 µL of Lipofectamine 2000 (Invitrogen, Carlsbad, CA) per well of a 6-well plate, according to manufacturer's instructions. Cells were then grown in medium containing 25 µg/ml mycophenolic acid (MPA), 250 µg/ml xanthine and 15 µg/ml hypoxanthine. MPA blocks the conversion of inosine monophosphate (IMP) to guanine monophosphate (GMP) affecting viral replication. The GPT enzyme is capable of circumventing this block if hypoxanthine and xanthine are present. Thus, only recombinant virus that contains the GPT gene (and Luc+) will form plaques. Recombinant viruses were plaque purified five times and assayed for luminescence in an IVIS imager (Caliper Life Sciences, Almeda, CA) in the presence of luciferin substrate (D-luciferin, Promega, Madison, WI).

### One-step growth curve

To determine whether the insertion of the luciferase gene into the intergenic regions affected the *in vitro* growth and other phenotypic characteristics of MPXV, we conducted one-step-growth studies using the MPXV-USA-2003, MPXV-USA-Luc+, MPXV-Congo, and MPXV-Congo-Luc+strains. Vero cells 0.5×10^6^/ml per well) were seeded in a 6-well plate and incubated overnight at 37C 5% CO_2_. Cell monolayers were infected with MPXV at a multiplicity of infection (MOI) 0.1 for 30 min and then washed twice with PBS and MEM media was placed onto the wells. At 0, 4, 12, 24, 48, 72, and 96 hours post-infection, three wells per virus strain were harvested (media and cells) by scraping and placed at −80°C. After three cycles of freezing and thawing, the samples were sonicated for three 20-second (sec) bursts of 75 W of output using a sonicator model W220 (Heat Systems Ultrasonics, Inc., Farmingdale, N.Y.) [38], and virus titers determined by serial dilution plaque assay on Vero cells. Plaques were visualized by staining with 0.1% crystal violet and virus titers determined as described elsewhere [39].

### Animal studies

All animal studies were conducted in the BSL-3 animal facility at the USGS-National Wildlife Health Center (NWHC, Madison, WI) and approved by the NWHC Institutional Animal Care and Use Committee (IACUC). Animal model development studies were conducted using 3-to 4 week-old female BALB/c and BALB/cJHanHad-Prkdc-SCID mice obtained from Harlan Sprague Dawley, (Indianapolis, IN). Groups of animals were inoculated by the intraperitoneal (IP, dose: 100 µl)) route, using either parental (MPXV-2003-USA-044, MPXV-Congo-Luc+), recombinant (MPXV-USA-Luc+, MPXV-Congo-Luc+) viruses, or PBS-control. Following viral infection, all animals were monitored twice a day for clinical signs and death. At different times post-infection mice were injected IP with 1.5 mg luciferin in 100 µl of DPBS (Promega, Madison, WI) and imaged in an IVIS 200 imager. Exposures for 30 sec (F8, medium binning) were taken at approximately 12 minutes post-luciferin injection following anaesthetization with isoflurane. An uninfected control animal was injected with luciferin and imaged simultaneously with infected animals. Images were analyzed with Living Image 3.0 software (Caliper Life Sciences, Alameda, CA). If death occurred or euthanasia was necessary to avoid animal suffering, tissue samples from spleen, liver, kidneys, intestines, ovaries, lung, heart, brain, and skin were aseptically collected and used for either measurement of virus titer (snap frozen at -70°C) or fixed in a 10% neutral buffered formalin solution.

### Immunohistochemistry and Histopathology

To determine whether biophotonic imaging correlated with the distribution of MPX viral antigen, immunohistochemistry (IHC) was used to detect MPXV antigen in tissue sections. At necropsy, brain, liver, kidney, heart, lung, intestine, adrenal glands, ovary and skin of the feet were collected from SCID/BALB/c mice infected with MPXV-USA-2003, MPXV-USA-Luc+, and from uninfected control SCID/BALB/c mice. These tissues were immediately fixed in 10% neutral buffered formalin. Tissues were sectioned at 5 µ and stained following standard IHC procedures using a vaccinia mouse hyperimmune ascitic fluid (1∶100 dilution, kindly provided by Dr. R. Tesh University of Texas Medical Branch, Galveston, TX) and HRP-conjugated anti-mouse IgG (Abcam, Cambridge, MA) as a detection label [18]. Tissue sections were also stained by the hematoxylin and eosin method.

### Titration of virus from mouse tissue samples

Tissues were weighed and homogenized in 500 µl of 1 mM Tris pH 9.0 in a pellet pestle homogenizer (Fisher Scientific, Pittsburgh, PA) followed by passage through a motorized Tissue Tearor (Biospec Products, Bartlesville, OK) for 15 sec. Between each tissue sample the homogenizer tip was sequentially submerged in 2% Bacdown viracide for 10 sec, 10% chlorox, 70% ethanol, and distilled water twice and then blotted dry. The homogenates were cleared by a 5 min centrifugation @ 6000×g in a tabletop centrifuge. For determination of virus titer, serial dilutions of each sample were made by mixing 45 µl of lysate in 405 µl of M199 media. Tubes were vortexed and four-fold serial dilutions prepared. Each dilution (100 µl) was plated in triplicate on a 48-well plate of 85% confluent Vero cells. The infection was incubated at 37°C for 5 hours followed by removal of the infection mixture and replacement with fresh M199 media. Plates were incubated four days and then stained with 5% crystal violet in 20% ethanol. Titer was calculated per gram of tissue and was compared between the wild type and Luc+ strain for each tissue using a t-test for unequal variances [40].

### Luminescent quantification of MPXV-Luc+ viruses

Tissue luminescence was quantified using the IVIS 2000 imager to develop a correlation curve between virus titer and luminescence for use in future studies to approximate virus titer. Briefly, tissue lysates (20 µl of 1∶10 v/v for kidney, liver, and lung; 1∶100 v/v for ovary) were added to an opaque black 96-well plate in triplicate (Fisher Scientific, Pittsburgh, PA), and incubated with 70 µl of a mixture containing 15 mg/ml luciferase substrate in sterile DPBS (Caliper Life Sciences, Hopkinton, MA), and the luminescence measured with the IVIS imager. Exposure time was adjusted to 30 sec to obtain the maximum unsaturated signal. A calibration curve was then generated using inverse regression analysis and plotting virus titer (PFU/g) against luminescence (photons/sec).
